# *DDX3Y* loss-of-function variant in AZFa-like non-obstructive azoospermia highlights gaps in current Y-chromosome microdeletion testing

**DOI:** 10.1530/RAF-26-0046

**Published:** 2026-10-06

**Authors:** Florence Abou, Netanel Waldenberg, Shany Kravat, Moran Gershoni, Sandra E Kleiman, Naama Steiner, Atef Zeadna, Guy Bar, Oshri Barel, Snir Dekalo, Ruti Parvari, Shlomi Barak

**Affiliations:** ^1^ Ben-Gurion University of The Negev, National Institute of Biotechnology in The Negev, Be’er Sheva, Israel; ^2^ Ben-Gurion University of The Negev, The Shraga Segal Department of Microbiology, Immunology and Genetics, Faculty of Health Sciences, Be’er Sheva, Israel; ^3^ The Multidisciplinary Center for Female and Male Infertility, Male Fertility, Tel Aviv, Israel; ^4^ Institute of Animal Science, Agricultural Research Organization, Volcani Center, Rishon LeZion, Israel; ^5^ Male Fertility Clinic and Sperm Bank, Lis Maternity Hospital, Tel Aviv Sourasky University Medical Center, Tel Aviv, Israel; ^6^ Gray Faculty of Medical and Health Sciences, Tel Aviv University, Tel Aviv, Israel; ^7^ Soroka University Medical Center, Fertility and IVF Unit, Dephlomiartment of Obstetrics and Gynecology, Be’er Sheva, Israel; ^8^ Samson Assuta Ashdod University Hospital, Reproductive Services, Ashdod, Israel; ^9^ Faculty of Health Sciences, Ben-Gurion University of The Negev, Be’er Sheva, Israel; ^10^ Department of Obstetrics and Gynecology, Samson Assuta Ashdod University Hospital, Ashdod, Israel; ^11^ Urology Department, Tel Aviv Sourasky Medical Center, Faculty of Medical Health and Sciences, Tel Aviv, Israel

**Keywords:** andrology, assisted reproduction, fertility, reproductive medicine, sperm

## Abstract

**Abstract:**

Non-obstructive azoospermia (NOA) due to spermatogenic failure is one of the most challenging conditions for surgical sperm retrieval (SSR). Complete AZFa deletions on Yq11 typically lead to a uniform Sertoli-cell-only (SCO) phenotype, rendering testicular sperm extraction futile. Current EAA/EMQN guidelines rely on sequence-tagged site (STS)-based PCR to detect multi-kilobase AZFa deletions; however, by design, they do not capture intragenic loss-of-function (LoF) variants in key AZFa genes. Recent exome studies have identified *DDX3Y* as the critical spermatogenic factor within AZFa, showing that isolated *DDX3Y* LoF variants can reproduce the classical AZFa-like SCO phenotype despite an apparently normal AZFa STS profile. We present a case of a man with idiopathic NOA and an AZFa-like phenotype; elevated FSH, normal karyotype, negative standard AZFa/b/c STS testing, and microdissection testicular sperm extraction (micro-TESE) showed complete SCO syndrome with no sperm retrieved. Whole exome sequencing (WES) subsequently revealed a novel likely pathogenic LoF variant in *DDX3Y* in one of its coding exons, a defect not detectable by routine AZFa PCR. This case adds to the small number of published *DDX3Y* loss-of-function variants associated with NOA and SCO syndrome and illustrates an important diagnostic limitation of STS-based AZF testing. We suggest that *DDX3Y* should be considered in exome or targeted-panel interpretation for selected men with NOA and an AZFa-like presentation despite negative microdeletion testing. Prospective cohorts are required before routine testing algorithms, SSR contraindications, or cost-effectiveness conclusions can be established. Upon validation, such an approach would avoid unnecessary, low-yield SSR while enhancing prognostic counseling and resource utilization.

**Lay summary:**

Some men have no sperm in the semen because sperm production in the testis has failed. In selected patients, surgical sperm retrieval may be performed, in which small pieces of testicular tissue are examined to look for rare sperm that could be used for intracytoplasmic sperm injection, a form of IVF in which a single sperm is injected into an egg. Before surgery, men are usually tested for large missing regions on the Y chromosome, including the AZFa region. Complete AZFa deletions are associated with a very poor chance of finding sperm. In this case report, we describe a man with no sperm in the semen, negative standard AZF deletion testing, and no sperm found during microdissection testicular sperm extraction. Later exome sequencing identified a previously unreported stop-gain variant in *DDX3Y*, a gene located within AZFa and now considered to have moderate evidence for involvement in severe sperm production failure. Together with previously published cases, this finding illustrates a limitation of standard deletion testing: it cannot detect small gene-level variants. In carefully selected men with an AZFa-like clinical picture, *DDX3Y* sequence analysis may improve counseling and may help avoid low-yield surgery, but larger studies and economic evaluation are needed.

## Introduction

Azoospermia is characterized by the complete absence of spermatozoa in the ejaculate, occurring in 10–15% of men undergoing fertility evaluations (Hwang *et al.* 2018). This must be confirmed through repeated tests involving vigorous centrifugation of the semen and meticulous examination of the pellet (WHO 2021). Non-obstructive azoospermia (NOA), which is associated with spermatogenic failure, is considered a particularly challenging form of azoospermia, typically managed by surgical sperm retrieval (SSR) followed by intracytoplasmic sperm injection. Microdeletions in the azoospermia factor (AZF) regions of the Y chromosome, specifically AZFa, AZFb, and AZFc, rank as the second most common genetic causes of NOA and are routinely assessed using multiplex PCR targeting sequence-tagged sites (STSs) (Krausz *et al.* 2024). Complete deletions of AZFa or AZFb invariably lead to azoospermia with distinctive testicular histology and virtually no chance of successful SSR, whereas AZFc deletions present a wider range from severe oligozoospermia to azoospermia, with SSR success rates between 50 and 60% (Kleiman *et al.* 2001, 2011, Jiao *et al.* 2024, Krausz *et al.* 2024, Krausz & Abrardo 2025). Current guidelines from the European Academy of Andrology (EAA) and the European Molecular Genetics Quality Network (EMQN) establish a robust STS-based framework for detecting these deletions and highlight the prognostic importance of accurately identifying complete AZFa and AZFb deletions (Krausz *et al.* 2024). However, this approach is inherently designed to capture only multi-megabase deletions and, crucially, fails to detect intragenic variants in key AZFa genes. These limitations underscore the importance of *DDX3Y*, which has recently been suggested to have an essential role in spermatogenic failure in AZFa, significantly reshaping clinicians’ understanding of this region (Dicke *et al.* 2023). This indicates that a negative AZFa PCR does not rule out an ‘AZFa-equivalent’ genetic cause for Sertoli-cell-only (SCO) syndrome.

## Objectives

This report provides an overview of the evolving understanding of AZFa and *DDX3Y*. It presents a clinically significant case in which standard AZF PCR failed to detect a *DDX3Y* defect, thereby further supporting the incorporation of targeted *DDX3Y* sequencing (or exome sequencing inclusive of *DDX3Y*) in the diagnostic evaluation of NOA. Additionally, this report examines the cost-effectiveness of this approach from a practical clinical perspective.

### AZFa, *DDX3Y*, and the limits of STS-based PCR

#### Classical view of AZFa

The absence of the complete AZFa region is caused by recombination between two provirus sequences spaced by 800 kb located in the proximal part of Yq11 (Kamp *et al.* 2000, Sun *et al.* 2000). The region contains three single-copy genes: *USP9Y, DDX3Y,* and *UTY* (Vogt *et al.* 2021). This rare deletion, found in 0.28% of NOA men (Kleiman *et al.* 2012), is considered to be associated with a grave prognosis and complete absence of germ cells in seminiferous tubules (SCO syndrome) (Krausz *et al.* 2024). In these men, SSR is very poor and not recommended (Krausz & Abrardo 2025). Numerous studies have described partial AZFa deletions, including the *USP9Y* or *DDX3Y* genes, or in part of each. The reported prevalence of partial AZFa deletions has a wide range (0.2–11%), and the histologic picture ranges from normozoospermia to SCO syndrome (Kleiman *et al.* 2012).

Deletion analysis of the AZFa region uses two STS markers, sY84 and sY86, located upstream of the *USP9Y* and *DDX3Y* genes, respectively. The absence of both markers strongly suggests a complete AZFa deletion. However, in rare cases, these markers are missing without affecting the AZFa genes or only affecting *USP9Y*. The deletion extension analysis presently uses five additional markers to verify the deletion of the AZFa genes, which are situated in the distal region (Krausz *et al.* 2024). While the STS approach detects large deletions, variations such as point mutations or small deletions in the AZFa genes are missed.

#### *DDX3Y* identified as the key AZFa gene

For decades, the question of which of the three AZFa genes is crucial for human spermatogenesis has remained unresolved (Dicke *et al.* 2023). Although initially considered a strong candidate, USP9Y has been shown to be non-essential since inherited deletions of *USP9Y* alone can coexist with normal spermatogenesis and fertility, suggesting a role in fine-tuning rather than being essential (Krausz *et al.* 2006, Luddi *et al.* 2009, Alksere *et al.* 2019). UTY encodes a histone demethylase with widespread expression and limited demethylase activity, and it shows no established infertility phenotype when disrupted individually (Shpargel *et al.* 2012, Vogt *et al.* 2021). These observations shifted attention toward *DDX3Y*, a DEAD-box Y RNA helicase, as a plausible gene-level contributor to the severe phenotype observed in complete AZFa deletions. Supporting this view, Dicke *et al.* screened whole exome sequencing (WES) data from over 1,600 men with unexplained cryptozoospermia or azoospermia for high-impact variants in *USP9Y, DDX3Y*, and *UTY* (Dicke *et al.* 2023). They identified four unrelated men carrying different loss-of-function (LoF) variants in *DDX3Y* (frameshift, nonsense, or splice site) that abrogated at least the C-terminal helicase domain. All four variants were absent from gnomAD (67,961 men), and one was confirmed to be *de novo*. Three of the four men underwent testicular sperm extraction, and all showed a pure SCO phenotype; the fourth had NOA with characteristic hormonal and testicular features but no biopsy. No comparable pathogenic variants were found in *USP9Y* or *UTY* in this cohort of patients. Functional data showing that germ cell formation defects in iPSC lines derived from men with AZFa deletions can be rescued by *DDX3Y* expression provide strong supportive evidence that DDX3Y loss of function can recapitulate an AZFa-like spermatogenic failure phenotype (Ramathal *et al.* 2015, Dicke *et al.* 2023).

Importantly, they demonstrated that loss of DDX3Y function alone is sufficient to cause an AZFa-like SCO phenotype. Such variants are not detected by standard AZFa STS PCR if the surrounding genomic region remains structurally intact.

## Methods

### Clinical evaluation and micro-TESE

The reported patient underwent a comprehensive infertility evaluation, including a detailed medical history, physical examination, at least two semen analyses, endocrine assessment, and scrotal ultrasonography. Semen analyses consistently demonstrated azoospermia according to the WHO 6th edition reference, with centrifugation performed and pellet examination confirming the absence of spermatozoa. Serum follicle-stimulating hormone (FSH) levels were elevated at 10.2 IU/L (reference range: 1–7 IU/L), while luteinizing hormone (LH) and testosterone (T) levels remained within normal ranges (7.4 IU/L and 18.8 nmol/L, respectively; reference range: LH: 2–10 IU/L and T: > 12 nmol/L). The testicular volume was reduced on the right side (11 mL) but was normal on the left (25 mL). Karyotyping revealed a normal 46,XY chromosomal pattern. Y-chromosome microdeletion analysis was performed in an accredited laboratory using multiplex PCR in accordance with the EAA/EMQN guidelines, targeting the recommended AZFa, AZFb, and AZFc STS markers (Krausz *et al.* 2024). No microdeletions were detected.

Microdissection testicular sperm extraction (micro-TESE) was performed under general anesthesia using an operating microscope to systematically inspect the testicular parenchyma and identify seminiferous tubules that appeared relatively dilated, more opaque, or larger in caliber than the surrounding tubules, in accordance with published techniques (Schlegel & Li 1998). The microdissection and tissue sampling phase lasted approximately 3 h, during which a total of 15 tissue samples were obtained from each testis. In parallel with the ongoing procedure, the initial sperm search was conducted for approximately 3 h using an inverted microscope at 200× magnification. When no spermatozoa were identified, an extended search was performed for an additional 10 h using three microscopes in parallel, including examination of processed tissue with an inverted microscope equipped with Nomarski optics, as previously described by Barak *et al.* (2025). No spermatozoa were identified in any of the examined samples, and sperm retrieval was therefore considered negative.

Representative testicular biopsy specimens were fixed in Bouin’s solution immediately after collection, routinely processed, paraffin-embedded, sectioned at 4–5 μm, and stained with hematoxylin and eosin (H&E) using the VENTANA HE 600 automated staining system (Roche Diagnostics, USA). Stained slides were subsequently digitized using the PANNORAMIC 480 Digital Slide Scanner (3DHISTECH Ltd, Hungary), and histopathological evaluation was performed on the resulting whole-slide digital images. Histological assessment included evaluation of overall testicular architecture, seminiferous tubule morphology, and spermatogenic activity according to predefined criteria, including seminiferous tubule integrity, tubular diameter, basement membrane thickening, germ cell composition and maturation, Sertoli-cell-only pattern, maturation arrest, hypospermatogenesis, tubular sclerosis/hyalinization, and interstitial alterations. All slides were independently reviewed by an experienced pathologist who was blinded to the clinical and laboratory findings to minimize observer bias, in accordance with established recommendations for rigorous histopathological reporting.

### Exome sequencing and variant interpretation

Whole exome sequencing was performed on genomic DNA extracted from peripheral blood. The sequencing was carried out at Theragen (South Korea). Exonic regions were enriched using a SureSelect XT Human All Exon Kit V6 + UTR, targeting approximately 60 Mb of the human genome with 738,690 probes. Sequencing was carried out on an Illumina NovaSeq X Plus platform using 151-bp paired-end reads that yielded an average coverage of >100X, and ≥90% of the target genome had a coverage of >X10.

Sequencing reads were aligned to the human reference genome (GRCh37/hg19). Variant calling was performed using the Genome Analysis Toolkit (GATK, version 4) following best practice guidelines. Variant call data were analyzed using Franklin by the Genoox platform (https://franklin.genoox.com/). Analysis was restricted to genes with established associations with human male infertility. Variants were evaluated within a custom gene panel associated with human male infertility, assembled from the literature and curated genomic databases, including OMIM (https://www.omim.org/), the Monarch Initiative (https://monarchinitiative.org/), Mouse Genome Informatics (MGI; https://www.informatics.jax.org/), and GeneCards (https://www.genecards.org/). Gene selection was further guided by previously published reviews of monogenic male infertility (Cioppi *et al.* 2021, Houston *et al.* 2021, Xavier *et al.* 2021). Variant filtering prioritized rare homozygous single nucleotide variants (SNVs) and small insertions/deletions (INDELs), with population allele frequencies <2% in public databases. Candidate variants of interest were validated by Sanger sequencing of the genomic regions containing the variants, using PCR amplification with primers flanking the target sites, to confirm the presence and zygosity of each variant. The primers sequences are provided in Supplementary Table 1 (see section on Supplementary materials given at the end of the article).

Local ethics approval and written informed consent for genetic testing and use of anonymized clinical data were obtained in line with institutional and national regulations.

## Results

No spermatozoa were retrieved during micro-TESE. Histological examination of the testicular tissue revealed a SCO pattern, with a complete absence of germ cells, indicative of an AZFa-like phenotype (Fig. 1A). As part of an exome study on idiopathic NOA, the patient underwent WES. This analysis identified a novel, likely pathogenic LoF variant in *DDX3Y* (((NM_004660.5), c.1501C>T, p.Arg501*), located in exon 14 of 17 coding exons; Fig. 1B). This nonsense variant likely introduces a premature termination codon (PTC) that is predicted to trigger nonsense-mediated mRNA decay (NMD) because it is located upstream of the final exon–exon junctions (Carrard & Lejeune 2023). If a transcript escapes NMD, translation will yield a truncated protein lacking the C-terminal helicase motifs essential for RNA binding and ATP hydrolysis, leading to loss of DDX3Y function (Fig. 1D). The variant is absent from population databases (gnomAD), providing additional support for its pathogenicity. In addition, population data from gnomAD indicate that *DDX3Y* is highly constrained for loss-of-function variation. In gnomAD v2.1.1, high-confidence predicted loss-of-function (pLF) variants are markedly depleted relative to expectation (observed/expected = 0.04; 90% CI: 0.01–0.12; pLI = 1), consistent with strong purifying selection against LoF in this gene. Across approximately 250,000 sampled Y chromosomes in gnomAD v2.1.1, only nine putative LoF alleles were observed, each ultra-rare (no more than a single allele in the dataset).

**Figure 1:**
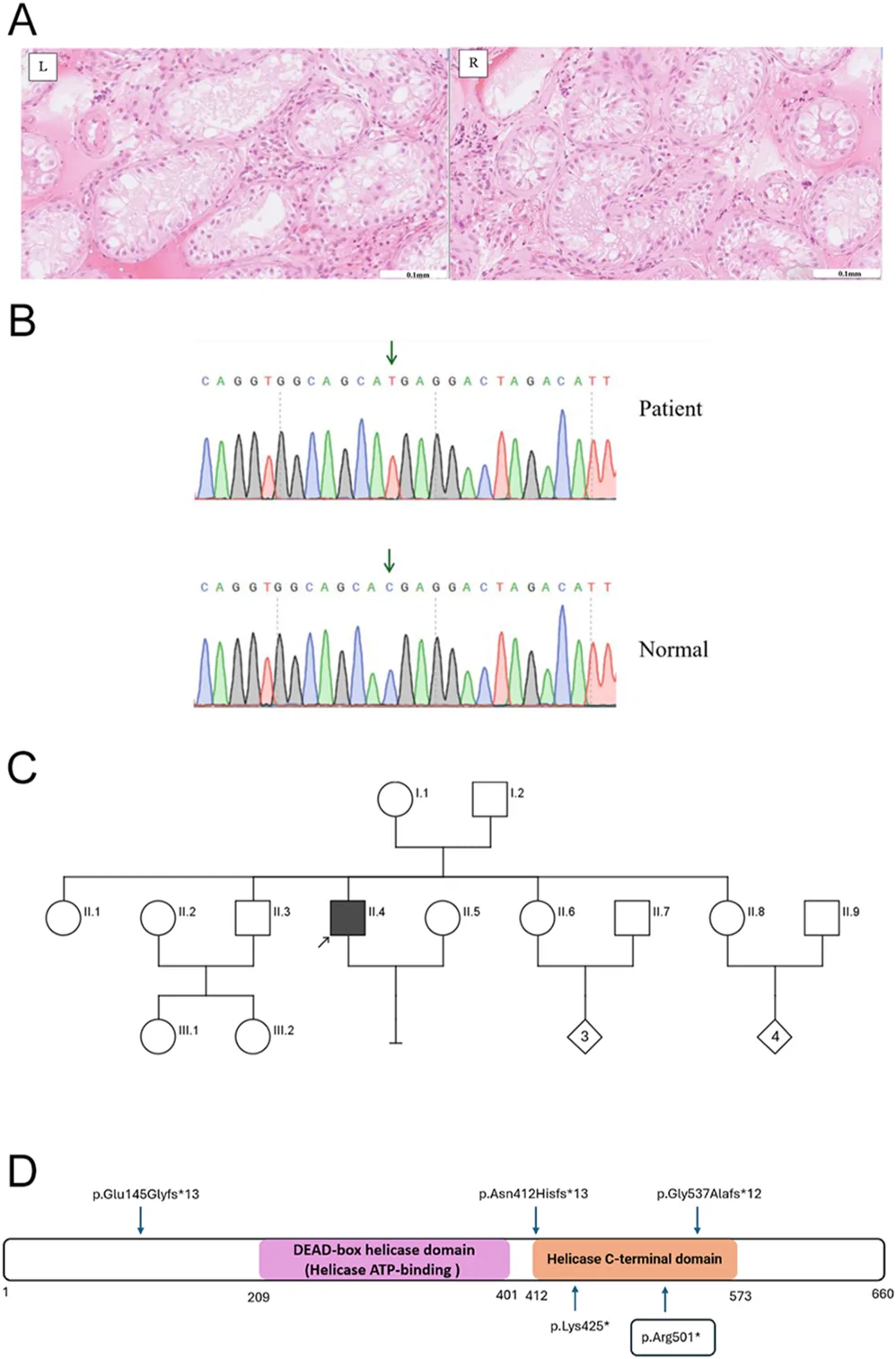
*DDX3Y* mutations. (A) Representative hematoxylin and eosin (H&E)-stained sections of the left (L) and right (R) testicular biopsies obtained from the affected individual. Images were acquired at 20× magnification (40× objective). Scale bar = 0.1 mm. (B) Sanger sequence chromatogram of the patient showing a homozygous variant NM_004660.5: c.1501C>T in the *DDX3Y* gene compared with the normal sequence. (C) Family pedigree of the reported family, generated using the CeGaT Pedigree Chart Designer. (D) Schematic representation of the DDX3Y protein with previously reported variants; the boxed variant represents the variant identified in our case.

As the variant is located on the Y chromosome and the patient’s brother is fertile (Fig. 1C), this finding argues against a shared inherited Y-linked defect and supports a likely *de novo* origin, although paternal germline mosaicism cannot be excluded. However, this hypothesis could not be tested because segregation analysis was not possible due to the unavailability of family members.

This variant was not covered by the routinely examined STS markers used in our Y-chromosome microdeletion PCR assay and was therefore completely overlooked by the standard AZFa testing. Thus, future validation of such AZFa-like *DDX3Y* LoF could offer a complementary genetic diagnosis of the predicted SCO phenotype and unrecommended micro-TESE for this patient.

## Discussion

The recent publication by Dicke *et al.* provides robust evidence supporting *DDX3Y* as a clinically relevant disease gene for NOA (Dicke *et al.* 2023). Analysis of WES data from 1,655 well-characterized men with unexplained crypto- or azoospermia identified four unrelated individuals carrying different LoF variants in *DDX3Y* (Table 1). Critically, the three patients who underwent TESE all showed SCO syndrome, the identical histological phenotype observed in complete AZFa deletions. This perfect genotype–phenotype concordance, as established by Dicke *et al.*, supports that DDX3Y LoF is a strong candidate to cause AZFa-like SCO phenotype. The presented patient had at least one testis with normal volume, unlike previously reported cases, in which all patients had reduced testicular volume (Table 1). If confirmed, this phenotypic variation in *DDX3Y* LoF variants represents a current limitation in our understanding of the full spectrum of DDX3Y-related pathology, suggesting possible differences in pathophysiology, genetic modifiers, or disease progression that warrant further investigation.

**Table 1:** Genotype–phenotype correlation in DDX3Y-related NOA: our patient (II-4 in this case) compared with published cases. SSR, surgical sperm retrieval; Neg, negative; T, testosterone; FSH, follicle-stimulating hormone; LH, luteinizing hormone. Reference normal range: TV: > 12 mL per testis, FSH: 1–7 IU/L, LH: 2–10 IU/L, and T: > 12 nmol/L.

Study/patient	Age	SSR histology	Sperm retrieval	cDNA alteration	Protein alteration	Variant type	Variant origin	FSH (IU\L)	LH (IU\L)	T (nmol\L)	Testicular volume (mL)	
											Right	Left
Dicke *et al.* (2023)												
M2171	31	SCO syndrome	Neg	c.1609+1del	p.Gly537Alafs*12	Splice site	*De novo* (proven)	18.1	4.9	10.58	9	12
M2185	37	SCO syndrome	Neg	c.1272dup	p.Lys425*	Nonsense	Unknown	6.1/9.8	6.7	22.14	7	9
M3086	34	SCO syndrome	Neg	c.428dup	p.Glu145Glyfs*13	Frameshift	Unknown	30.9	5.3	8.23	10	10
Nagirnaja *et al.* (2022)												
GEMINI-492	43	No biopsy performed	Neg	c.1230_1231del	p.Asn412Hisfs*13	Frameshift	Unknown	27.2	9.3	8.4	10	10
II-4 presented case	26	SCO syndrome	Neg	c.1501C>T	p.Arg501*	Nonsense	*De novo* (suspected)	10.2	7.4	18.8	11	25

*indicates a premature stop codon according to HGVS nomenclature.

### Cost-effectiveness and practical considerations

From a laboratory perspective, classical AZF deletion screening by multiplex PCR is relatively simple, rapid, and inexpensive and can be implemented in most routine molecular laboratories (Krausz *et al.* 2024). It is well suited for detecting large AZFa, AZFb, and AZFc deletions and, when correctly performed, provides robust prognostic information: complete AZFa or AZFb deletions predict SSR failure, whereas AZFc deletions indicate a roughly 60% chance of sperm retrieval (Jiao *et al.* 2024).

The present case illustrates a limitation of this region-based testing strategy. STS-based AZF testing interrogates selected genomic landmarks and is not designed to detect sequence-level variants, small insertions or deletions, or single-exon alterations within individual AZFa genes, such as *DDX3Y*. Therefore, a normal AZFa STS result does not exclude a gene-level defect affecting an AZFa candidate gene. In this patient, standard AZF testing was negative, whereas exome sequencing subsequently identified a rare stop-gain variant in *DDX3Y*, in association with a SCO histological phenotype and failed sperm retrieval.

Although this observation is consistent with the four previously reported men carrying *DDX3Y* loss-of-function variants, the available evidence remains limited to a small number of cases. Therefore, the clinical implications should be interpreted cautiously. At present, our findings should not be taken as sufficient evidence to mandate routine *DDX3Y* sequencing for all men with NOA, nor to establish a universal contraindication to surgical sperm retrieval in every individual carrying a *DDX3Y* variant. Rather, this case supports the view that *DDX3Y* should be considered in the genetic interpretation of selected men with idiopathic NOA, particularly when the phenotype resembles complete AZFa-associated spermatogenic failure despite negative STS-based AZF testing.

From a practical perspective, targeted sequencing or exome-based analysis may be valuable in this selected clinical context. Micro-TESE is an invasive procedure that requires surgical, anesthetic, histopathological, and embryology resources, and failed retrieval may impose a substantial emotional and financial burden on couples. However, because no formal health economic analysis was performed in the present study, we cannot conclude that *DDX3Y* sequencing is cost-effective. Instead, our case suggests that future studies should evaluate whether incorporating *DDX3Y* into targeted NOA gene panels or exome interpretation pipelines could improve preoperative counseling, reduce low-yield procedures in genetically defined subgroups, and support more individualized decision-making.

### Practical implications for clinicians

- **The STS PCR, which detects deletion of the AZFa on chromosome Y, does not exclude an AZFa-equivalent defect.** LoF variants in *DDX3Y* can cause classic SCO syndrome, despite intact STS markers.
- ***DDX3Y* should be considered a clinically relevant candidate gene in selected cases of idiopathic NOA.** Current evidence supports an association between DDX3Y loss of function and severe spermatogenic failure, but the number of reported cases remains small, and additional genotype–phenotype data are needed.
- **SSR counseling should incorporate uncertainty.** The reported cases suggest that men with loss-of-function *DDX3Y* variants and an AZFa-like phenotype may have a very poor probability of sperm retrieval. However, prospective validation is required before *DDX3Y* findings alone can be used as an absolute basis to discourage SSR.
- **A stepwise diagnostic framework may be considered for future evaluation.** We propose the diagnostic algorithm in Fig. 2 as a preliminary framework rather than as a validated clinical guideline. In this framework, standard clinical evaluation, karyotyping, and AZF microdeletion testing remain first-line investigations. In men with negative AZF testing but strong clinical or histological features suggestive of AZFa-like spermatogenic failure, *DDX3Y* analysis through a targeted-panel or exome sequencing may be considered as part of an expanded genetic evaluation. Upon identification of *DDX3Y* LoF, SSR may not be recommended.
- **Broader exome-based diagnostics may help define clinically actionable subgroups.** As genome sequencing becomes more widely used in the evaluation of severe male infertility, systematic reporting of *DDX3Y* and other spermatogenic failure genes will be important for refining genotype–phenotype correlations, estimating sperm retrieval outcomes, and determining whether specific genetic findings should influence surgical decision-making.

### NOA’s monogenic reality: single cases lay the groundwork for evidence evolution

Although a single observation, this *DDX3Y* report adds to 4 prior LoF variants (Dicke *et al.* 2023), cumulatively strengthening the gene–disease association toward strong evidence. We stand in an intermediate stage where most disease genes rely on small numbers of unrelated probands, often with variable histology and incomplete SSR follow-up. Such cases lay the groundwork for future consensus curation, transforming sparse reports into validated risk modifiers or SSR contraindications. Given the current lack of, or limited, experimental tools for validating genetic findings in male infertility, recurrence of genes and variants has become an important and powerful approach for untangling the genetic complexity of infertility. This report also catalyzes the ethical dialogue: should *de novo* DDX3Y p.Arg501* now contraindicate futile SSR, exome/targeted sequencing can spare unnecessary surgical interventions while catalyzing precision medicine in andrology.

**Figure 2:**
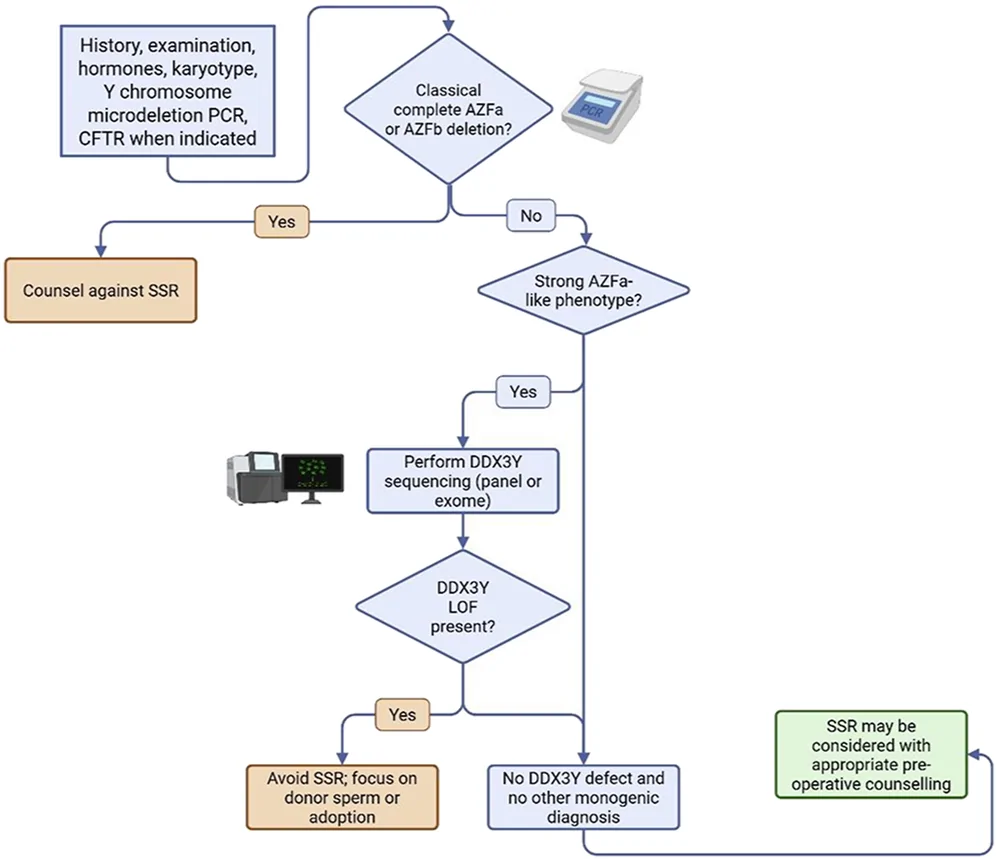
A proposed stepwise diagnostic algorithm for NOA.

## Conclusion

The identification of a rare truncating *DDX3Y* variant in this study adds to the growing number of reports linking DDX3Y loss of function with severe spermatogenic failure and an AZFa-like phenotype. This case highlights an important diagnostic limitation of STS-based AZF testing, namely that sequence-level defects in individual AZFa genes are not detected by routine microdeletion assays. However, because the current evidence is based on a limited number of cases and lacks prospective validation, the clinical use of *DDX3Y* sequencing should be framed cautiously. Incorporating *DDX3Y *into targeted NOA panels or exome-based interpretation may improve genetic diagnosis and preoperative counseling in selected men with idiopathic NOA, but its effect on surgical decision-making and cost-effectiveness should be evaluated in larger cohorts.

## Supplementary materials



## Declaration of interest

The authors declare that there is no conflict of interest that could be perceived as prejudicing the impartiality of the work reported.

## Funding

This work was supported by the Israel Science Foundation (ISF; Grant No. 2844/21).

## Author contribution statement

FA, RP, and SB conceived this study. They all contributed equally to this work. FA and RP performed the genetic and bioinformatic analyses and contributed to the literature review, editing and intellectual content of the manuscript, and preparation of tables and figures. SB contributed to patient recruitment, drafting of the manuscript, literature review, and preparation of tables and figures. NW and SK were responsible for patient recruitment, clinical data collection, and study administration. MG and SEK shared their database and contributed to the editing and intellectual content of the manuscript. NS and AZ contributed to the study design, administration, and coordination. GB, OB, and SD assisted with patient recruitment and contributed to manuscript editing and proofreading. All authors critically reviewed the manuscript, approved the final version, and agreed to be accountable for the accuracy and integrity of the work.
