# Supplementary material for: DDX3Y loss-of-function variant in AZFa-like non-obstructive azoospermia highlights gaps in current Y-chromosome microdeletion testing
Source: Reprod Fertil. 2026 Oct 6;7(4):RAF260046. doi: 10.1530/RAF-26-0046 (PMC13695432; doi:10.1530/RAF-26-0046)
Supplement: Supplementary file 1 [file RAF-26-0046_supplementary_table_1.pdf]

Supplementary Table 1

Primer	sequence
**Forward-DDX3Y**	GGAGACCGGTCACAGAGAGAT
**Reverse-DDX3Y**	TGAACTAACAAACCCCTTACCCA
